# Water-Tree Resistability of UV-XLPE from Hydrophilicity of Auxiliary Crosslinkers

**DOI:** 10.3390/molecules25184147

**Published:** 2020-09-10

**Authors:** Jun-Qi Chen, Xuan Wang, Wei-Feng Sun, Hong Zhao

**Affiliations:** Key Laboratory of Engineering Dielectrics and Its Application, Ministry of Education, School of Electrical and Electronic Engineering, Harbin University of Science and Technology, Harbin 150080, China; chenjunqi@hrbust.edu.cn (J.-Q.C.); topix_xuan@sina.com (X.W.)

**Keywords:** water tree, auxiliary crosslinker, photon-initiated crosslinking reaction, mechanical property

## Abstract

The water-resistant characteristics of ultraviolet crosslinked polyethylene (UV-XLPE) are investigated specially for the dependence on the hydrophilicities of auxiliary crosslinkers, which is significant to develop high-voltage insulating cable materials. As auxiliary crosslinking agents of polyethylene, triallyl isocyanurate (TAIC), trimethylolpropane trimethacrylate (TMPTMA), and N,N′-m-phenylenedimaleimide (HAV2) are individually adopted to prepared XLPE materials with the UV-initiation crosslinking technique, for the study of water-tree resistance through the accelerating aging experiments with water blade electrode. The stress–strain characteristics and dynamic viscoelastic properties of UV-XLPE are tested by the electronic tension machine and dynamic thermomechanical analyzer. Monte Carlo molecular simulation is used to calculate the interaction parameters and mixing energy of crosslinker/water binary systems to analyze the compatibility between water and crosslinker molecules. Water-tree experiments verify that XLPE-TAIC represents the highest ability to inhibit the growth of water-trees, while XLPE-HAV2 shows the lowest resistance to water-trees. The stress–strain and viscoelastic properties show that the concentration of molecular chains connecting the adjacent lamellae in amorphous phase of XLPE-HAV2 is significantly higher than that of XLPE-TAIC and XLPE-TMPTMA. The molecular simulation results demonstrate that TAIC/water and TMPTMA/water binary systems possess a higher hydrophilicity than that of HAV2/water, as manifested by their lower interaction parameters and mixing free energies. The auxiliary crosslinkers can not only increase the molecular density of amorphous polyethylene between lamellae to inhibit water-tree growth, but also prevent water molecules at insulation defects from agglomerating into micro-water beads by increasing the hydrophilicity of auxiliary crosslinkers, which will evidently reduce the damage of micro-water beads on the amorphous phase in UV-XLPE. The better compatibility of TAIC and water molecules is the dominant reason accounting for the excellent water resistance of XLPE-TAIC.

## 1. Introduction

With the rapid development of the power energy industry and the expansion of urban scale, the demand for power cables is significantly increasing and they required to be adapted to the gradually expanding application scope. The performances of the cable insulation layer, as one of the pivotal parts of power cable, determines the transmission capacity and service conditions of power systems. Crosslinked polyethylene (XLPE) has the advantages of high temperature resistance, excellent dielectric strength, and favorable mechanical properties, and is the most commonly used material for the insulation layer in power cable [1]. In wet environments, the micro-water beads around the power cable will gradually penetrate into the XLPE insulation layer and form water-trees under an electric field [2]. Although the formation of water-trees will not immediately cause cable insulation failure, water-trees will accumulate and continue expanding inside the insulation layer over the operation time, resulting in the increase of dielectric loss, the reduction of dielectric breakdown voltage, and even the thorough damage of cable insulation [3]. Water-tree aging is one of the primary factors leading to the dielectric deterioration of XLPE insulation, especially for medium voltage power cables. Therefore, for the XLPE insulation of a medium voltage power cable, it should thereby be prudent to pay more attention to the capable resistance to water-tree aging in addition to the necessary requirements of mechanical, electrical, and heat-resistant performances. The inception and growth of water-trees rely not only on the external factors, such as electric field strength and frequency, aging time, ambient temperature, but also on the internal factors such as crystallization morphology, and type and content of additives in the insulating materials [4,5,6,7]. In order to efficiently ameliorate the water resistance of the cable insulation layer, the representative technology of rejuvenation liquid injection has been developed to restrict the insulation aging from water-trees growth [8,9]; whereas the inadequate diffusion of rejuvenation liquid in polyethylene materials is technologically inevitable for the insulated cable applications.

At present, the main schemes to improve the water-tree-resistant performance of XLPE comprise the polar compound modification, filling with nanoparticles (nanodielectrics), adding elastomer, and using water-resistant materials [10,11,12,13]. The modification methods of nanodielectrics and adding elastomer are still in the laboratory research stage due to the limitation of technological conditions. The polar compound modification can inhibit the agglomeration of water microbeads at insulation defects, thus reducing the developing speed of water trees, which, however, cannot ensure that the cable has good water-resistance in long time operation due to the high probability of the molecule outgoing migrations caused by the substantial discrepancy in the polarities of the added polar compounds and XLPE. Although there is still no a general conclusion on the aging mechanism of polymer water-tree resistance, the electromechanical model of the water-tree growth mechanism is now comprehensively accepted [14]. According to the electromechanical mechanism of water-tree growth in polymer materials, the mechanical force produced by water molecules under alternating electric field will continue impacting on the macromolecular chains to finally form water-filled microcracks of tree structure in polymer amorphous area. Both linear polyethylene and XLPE are semicrystalline polymers, which are composed of crystalline and amorphous phases. The crystalline phase has complete lattices with a relatively high stability compared with the amorphous phase, thus water-trees tend to grow in the amorphous phase. Due to the difference of dielectric constant between water and polymer, electrostriction will occur in the water molecules under electric field, leading to local pressure (especially at the tip of defect) in the amorphous phase. This pressure will cause cracking in the material as well as the initiation and growth of water-trees [15]. Under a high electric field with high frequency, the water microbeads render the electrical and mechanical stresses on the macromolecules in the amorphous phase and eventually cause the molecular chains between polyethylene lamellae to be broken due to stress fatigue, after which fine cracks will appear in the amorphous phase. Consequently, water molecules are squeezed into cracks to form water-filled channels which will gradually expand under alternating electric field [16].

The ultraviolet-initiated polyethylene crosslinking process is a new power cable manufacturing technology developed in recent years, the principle of which is to make use of the photon-initiators to form free radicals by irradiating ultraviolet (UV) light through the transparent polyethylene insulating layer which is just extruded out in a melting state. The hydrogen atom in polyethylene macromolecular chains is captured by photon-initiators after being excited by UV irradiation, and then the bonding bridges are generated between polyethylene macromolecular chains to form three-dimensional network structures [17,18,19]. The UV-initiated polyethylene crosslinking technology, with the advantages of high crosslinking speed, low investment cost in small area, and long continuous processing time, presents multiple selectivity of auxiliary crosslinkers to develop polyethylene-based cable materials with excellent insulation performances. So far, the UV-initiated polyethylene crosslinking process has been successfully used in the production of 10 kV voltage grade power cables, with the prospective potential to be applied for manufacturing higher voltage grade power cables [20]. Compared with the traditional chemical method employing thermal decomposition of peroxide, the requirements of the pristine polyethylene and extrusion temperature of insulating layer are lower in the UV crosslinking process. Especially, the polyethylene insulation layer will not cross-link in an extruder, thus the auxiliary crosslinkers commonly used in industry can be utilized to accelerate the reaction rate of polyethylene crosslinking reaction. The auxiliary crosslinkers can not only greatly improve the crosslinking reaction rate of polyethylene, but also introduce charge traps with deep energy level into polymer electronic states, resulting in space charge suppression and high DC breakdown strength [21,22]. Because the auxiliary crosslinkers have formed chemically bonding bridges between polyethylene macromolecular chains, the crosslinker molecules will not migrate out when the cable is hot-degassed in vacuum and the XLPE insulation layer can persist in a stable electrical performance for a long time.

Polar compounds can be used as modifying agents to improve water resistance and insulation performances of XLPE. Because the auxiliary crosslinkers used in UV-initiated polyethylene crosslinking reactions belong to polar molecules as water, the hydrophilicity of crosslinkers will inevitably affect the diffusion behavior of micro-water beads at insulation defects, and thereby substantially determine the water resistance of UV-initiated XLPE (UV-XLPE). Therefore, it is of engineering significance and scientific value to study the hydrophilicity of auxiliary crosslinkers that affect the water resistance of UV-XLPE. In practice, it should take several years to observe the initiation and growth of water-trees in the cable insulation layer. The accelerating experimental method of water-tree aging is generally used to evaluate the water resistances of polymer materials. For the traditional water needle electrode method, a considerable number of water-trees often grow around the needle rod due to the small curvature radius of needle tip, which disturbs the evaluation of water-tree aging experiments. Therefore, the water blade electrode method [23,24] is adopted to test the water resistances of XLPE materials prepared with different auxiliary crosslinkers.

## 2. Results and Discussion

### 2.1. Crosslinking Degree and Water-Tree Morphology

As shown by the thermal elongation and gel content listed in Table 1, under the same mass fraction, the XLPE sample prepared by using HAV2 as auxiliary crosslinking agent acquires the highest crosslinking degree. This result is attributed to the relatively lower energy required for HAV2 to form free radicals during the crosslinking reaction, which makes is easier for BP to seize hydrogen atoms and for HAV2 to form bridge bonds with polyethylene macromolecular chains.

Due to the statistic dispersion of water-tree dimension, the 2-parameter Weibull statistics is implemented to evaluate water-tree structures by fitting experimental results with the statistic distributions as follows [16],

(1) $$F\left(L\right)=1-\exp\left[-{\left(\frac{L}{L_{s}}\right)}^{\beta }\right]$$

where *L* denotes water-tree size (length or width) for a slice sample; *L_s_* represents the characteristic value of water-tree dimension with a probability of 63.2%; *β* signifies the shape parameter indicating the dispersivity of experimental data; and *F*(*L*) symbolizes the failure probability of the water-tree structure with a dimension less than or equal to L, expressed as

(2) $$F\left(L,n\right)\approx \frac{i-0.3}{n+0.4}\times 100\%$$

where *i* denotes the number in ascending order of the tested samples and *n* is the total number of the tested samples for the same material, which is equal to 10 for the present experiments.

**Figure 1:**
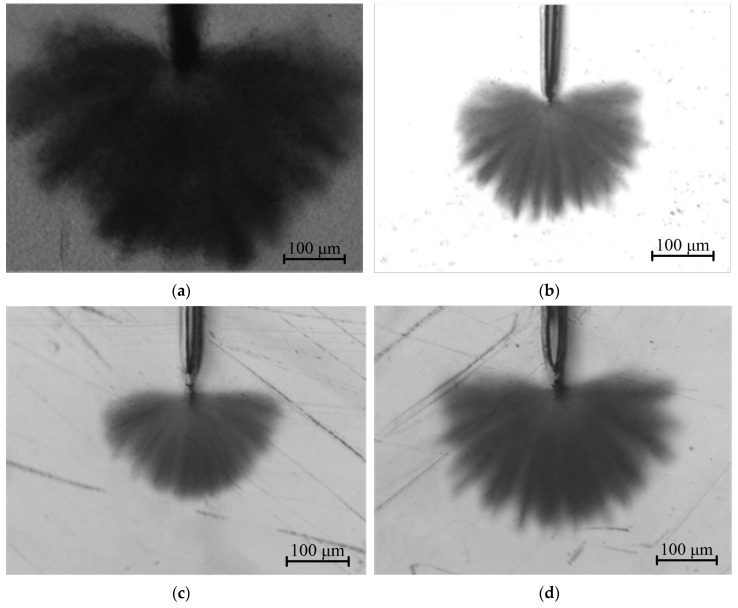
Water-tree morphology: (**a**) LLDPE, (**b**) XLPE-TMPTMA, (**c**) XLPE-TAIC, and (**d**) XLPE-HAV2.

As shown by the water-tree morphology of the LLDPE and UV-XLPE with different auxiliary crosslinkers in Figure 1, water-tree growth in LLDPE is not uniform in different directions, as manifested by the prominent branches around water-tree area, while the water-tree structures of UV-XLPE samples show a relatively more uniform shape with smaller branches. The polyethylene macromolecular chains will form a network structure after crosslinking reactions, which can disperse the impacting forces from water microbeads and thereby restrict the stresses formed under alternating electric field from being concentrated on local region.

**Figure 2:**
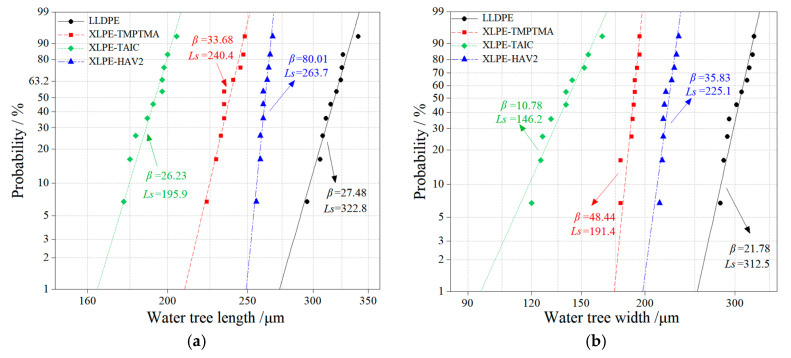
Weibull 2-parameter distributions fitting on the (**a**) length and (**b**) width of water-trees.

The water-tree growth can be evaluated by the characteristic length and width of water-tree from Weibull distributions, the lower value of which indicates a higher resistance to water-trees, as shown by the experimental results fitted by 2-parameter Weibull statistics with the characteristic value *L_s_* and shape parameter *β* being remarked in Figure 2. While shape parameter *β* characterizes the dispersion of experimental data, it is considered from the remarkable difference in the sizes of water-trees between the samples that the calculated shape parameters do not influence the judgment on water resistance. Therefore, we do not analyze the shape parameters in the present paper. It is indicated from Figure 1 and Figure 2 that the water-tree dimension of LLDPE is much larger than that of UV-XLPE, implying that all the three auxiliary crosslinkers can be used to appreciably ameliorate the water-tree resistance of polyethylene. With the identical mass ratio of auxiliary crosslinkers, XLPE-TAIC shows the lower values of characteristic length and width of water-trees than that of the other two UV-XLPE, especially being decreased by 26% and 35% in characteristic length and width, respectively, compared with XLPE-HAV2. The previous studies showed that the higher water-tree resistance (smaller water-tree size) arises in tandem with a higher crosslinking degree, which is not consistent with our results. The crosslinking degree of XLPE-HAV2 is higher than that of XLPE-TAIC and XLPE-TMPTMA, while the water-trees produced in XLPE-HAV2 are evidently smaller than that formed in XLPE-TAIC and XLPE-TMPTMA. In particular, XLPE-TAIC presents the lowest crosslinking degree in accompany with the highest water-tree resistance compared with the other two UV-XLPE.

### 2.2. Viscoelastic Properties

**Figure 3:**
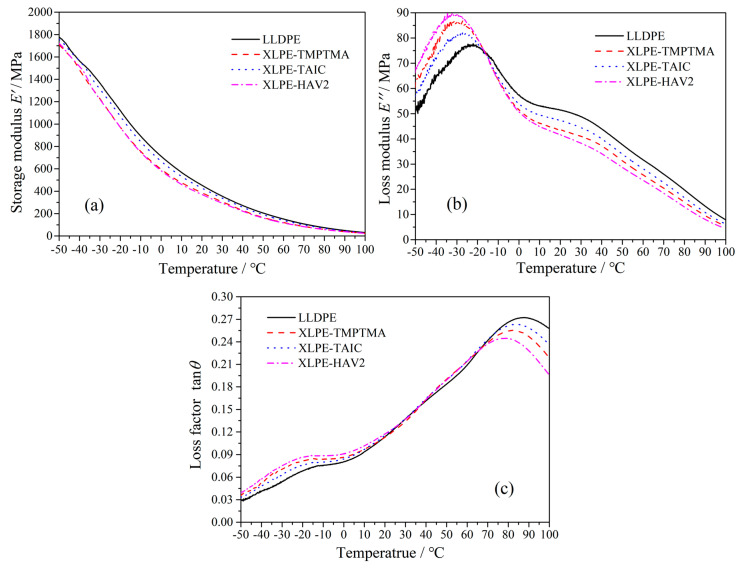
Dynamic thermomechanical analysis (DMA) temperature spectra of LLDPE and UV-XLPE: (**a**) storage modulus, (**b**) loss modulus, and (**c**) loss factor.

The dynamic relaxation temperature spectra of the storage modulus *E*′ implying the stored energy by elastic deformation, the loss modulus *E*″ identifying the energy loss in form of heat caused by viscous deformation, and the loss factor tan*θ* (ratio of *E*″ to *E*′; *θ* denotes the plural angle of complex modulus) signifying the varying trend of polymer viscoelastic properties are, respectively, shown in Figure 3a–c. The kinetic activity of molecular chains will be expedited by increasing temperature, leading to reduction in material rigidity as manifested by the declining elastic modulus *E*′. Generally, a higher crosslinking degree will result in a lower storage modulus of XLPE, which is consistent with the result that the highest and lowest *E*′ have been acquired by XLPE-TAIC and XLPE-HAV2, respectively. The loss modulus *E*″ increases first and then decreases with the increase in temperature, in contrast to the monotonously declining storage modulus *E*′ with increasing temperature. The loss modulus peak of polyethylene at approximately −30 °C marks the glass transition process and is called the *β* peak, which derives from the relaxation motions of the molecular chains being restricted between lamellae of polyethylene [25,26]. As the crosslinking degree of polyethylene increases, the *β* peak shifts to low temperatures and the amplitude rises gradually, implying that the molecular relaxations are exacerbated [27]. The loss factor peak arising at the relatively higher temperature is called *α* peak, which indicates the mechanical relaxations originating from the rotation and slip of the folded molecular chains in polyethylene lamellae [28]. The crosslinking reaction can increase the concentration and thus decrease the relaxation motions of molecular chains in amorphous area between polyethylene lamellae, and simultaneously impede the rotation and slip of the folded chains on lamella surface, as indicated by the highest *β* and the lowest *α* relaxation peaks of XLPE-TAIC.

As the tenacity of polymer materials is mainly reflected in the absorption of external forces, the loss modulus *E*″ and loss factor tan*θ* at the glass transition temperature can be used to measure the tenacity of the amorphous phase in polyethylene materials. The peak values of loss modulus and the corresponding loss factors in correlation with water-tree dimensions for LLDPE and UV-XLPE are listed in Table 2. It is noted that UV-XLPE materials prepared with each auxiliary crosslinker represent higher *E*″ and tan*θ* than LLDPE, indicating that a higher tenacity has been acquired after crosslinking reactions to reduce the impacts from water microbeads. However, the highest *E*″ and tan*θ* arise in the UV-XLPE material with a relatively lower water-tree resistance, while XLPE-TAIC with a relatively lower *E*″ and tan*θ* shows the highest water-tree resistance. It is thus clearly suggested that the water resistance of UV-XLPE prepared with auxiliary crosslinker cannot be comprehensively elucidated only by analyzing viscoelastic properties.

### 2.3. Stress–Strain Characteristics

**Figure 4:**
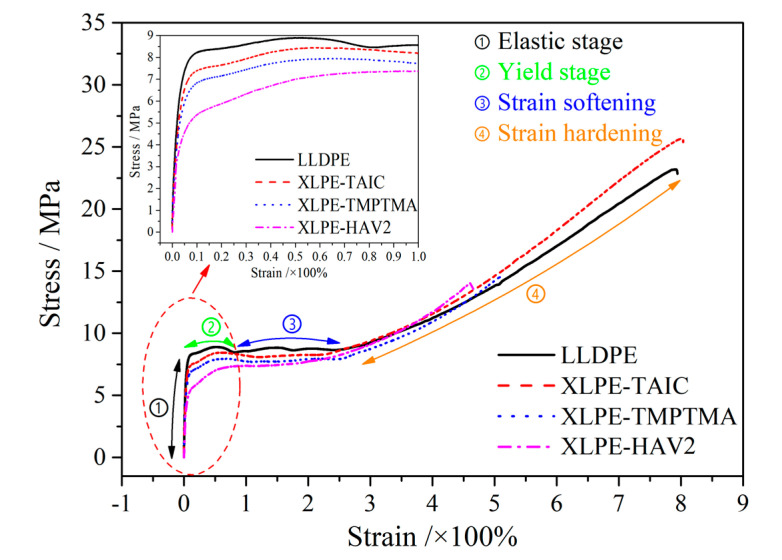
Stress–strain characteristics of LLDPE and UV-XLPE.

Stress–strain curves characterize the mechanical tensile processes consist of elastic, yield, strain softening, and strain hardening stages, as shown in Figure 4. Except for XLPE-TAIC, the UV-XLPE samples show significantly lower break stress and elongation at break than LLDPE, due to the considerable breakages of macromolecular chains caused by the excessive crosslinking reactions [29,30]. Both the elastic modulus and yield strength of polyethylene are reduced by crosslinking reactions, with the strain softening and cold drawing processes fading away, as shown in comparison of LLDPE and XLPE samples. In particular, for XLPE-HAV2, the elastic stage directly transits to the cold drawing stage without strain softening process. The crosslinking reaction produces more connecting molecular chains between the lamellae, which inhibits the slips between lamellae and thus abates the strain softening and cold drawing processes.

The polyethylene macromolecular chains extending through several crystalline regions in XLPE will be mutually entangled to each other. Therefore, there is a large number of molecular chains connecting lamellae in amorphous phase and the chain tails and rings of polyethylene molecules that have been excluded from crystalline phase of XLPE, which will suppress the slips between lamellae and be apt to suffer impacts from water microbeads under electric field, respectively. When the semicrystalline polymer is in the strain hardening stage during the tensile process, the amorphous phase will be “tighten”, where the molecular chains connecting molecule crystal will bear the majority of mechanical forces. A higher concentration of the connecting molecular chains in amorphous phase requires a higher external force for the identical deformation in the mechanical tensile process. Therefore, it is feasible to characterize the molecule density of amorphous phase by the gradient of stress–strain curves in strain softening stage.

It is proved from the water-tree dimensions of UV-XLPE listed in Table 2 that there is no positive correlation between strain hardening strength and water-tree size. While the UV-XLPE prepared with auxiliary crosslinker definitely shows higher water-resistance and strain hardening strength than LLDPE, XLPE-HAV2 with the highest strain hardening strength represents the lowest water-tree resistance compared with the other UV-XLPE materials. Thus, merely analyzing stress–strain characteristics, we cannot fully comprehend the water resistance of UV-XLPE prepared by using different auxiliary crosslinkers.

### 2.4. Miscibility of Crosslinker and Water

**Figure 5:**
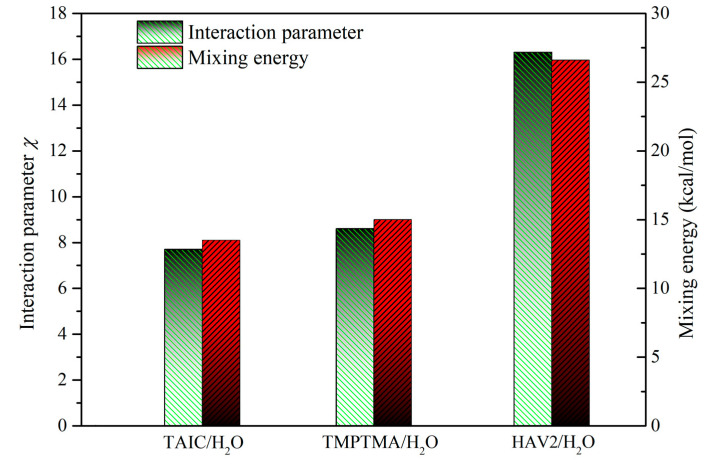
Mixing energies and interaction parameters of the binary systems comprising molecules of water and auxiliary crosslinker at room temperature.

Monte Carlo molecular simulations have been performed for the binary systems of TAIC/H_2_O, TMPTMA/H_2_O, and HAV2/H_2_O to evaluate the miscibility of crosslinker and water. The TAIC/H_2_O and TMPTMA/H_2_O binary systems show remarkably lower mixing energy *E*_mix_ and interaction parameter *χ* than the HAV2/H_2_O binary system at room temperature, as shown in Figure 5. In general, a small or negative value of *χ* indicates that the two molecules have a favorable interaction at a particular temperature and the mixture of two components will show just one phase. If *χ* is large and positive, the molecules prefer to be surrounded by similar components rather than each other. If the *χ* value is high enough so that the free energy overcomes the combinatorial entropy, the mixture of two components will separate into two phases. The higher *χ* and *E*_mix_ values indicate less miscibility of the pair molecules. Thus, water molecules are easier to dissolve and diffuse in TAIC and TMPTMA than in HAV2. Further, TAIC possesses the highest H_2_O solubility compared with the other two auxiliary crosslinkers. Taking into account that the crosslinkers have been grafted by form bridge bonds onto the macromolecular chains of polyethylene, it is reasonably suggested that the infiltrating water molecules are prone to form the dispersed small water droplets and will not agglomerate into large water beads in XLPE-TAIC and XLPE-TMPTMA. As consistently verified by the water-tree dimensions shown in Figure 1 and Figure 2, the lowest size of water-tree growing in XLPE-TAIC is coincident with the highest miscibility of TAIC/H_2_O.

**Figure 6:**
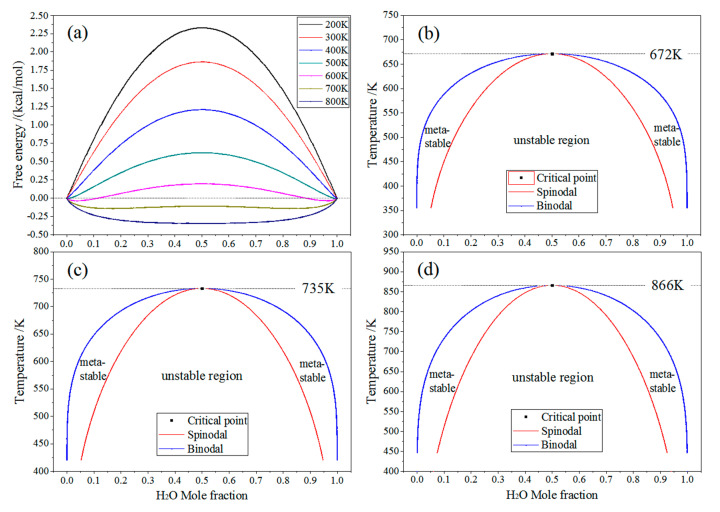
(**a**) The free energy of mixing for TAIC/H_2_O mixture varying with mole fraction for 200–800 K temperatures; mixture phase diagrams of (**b**) TAIC/H_2_O, (**c**) TMPTMA/H_2_O, and (**d**) HAV2/H_2_O binary systems.

The phase diagram, as a useful demonstration illustrating the compatibility of binary mixtures, generally comprises three pieces of information: critical points, binodals, and spinodals, as shown in Figure 6b,c. At the critical point, both the second derivative and third derivatives of the free energy with respect to composition vanish. The coexistence region is bound by the binodal, in which the mixture can lower its free energy by separating into two phases. The coexistence region also gives rise to two compositions where the second derivative of the free energy is zero, as shown in Figure 6a. The line through these points is called the spinodal, which separates the coexistence region into two regions: the mixture is metastable only when phase start separating after a sufficiently large fluctuation between the binodal and spinodal, or the mixture is unstable in the region bounded by the spinodal, and thus any fluctuation will cause the mixture to spontaneously separate. Although H_2_O solubility in TAIC or TMPTMA is rather small, there are substantial metastable regions between spinodal and binodals in phase diagram presenting significantly higher H_2_O molecule concentrations than that in HAV2, as illustrated in Figure 6. Compared with TMPTMA and HAV2, the lowest critical temperature (672 K) of the TAIC/H_2_O binary system at the coordinate blending point of mole ratio 1:1 in phase diagram implies the highest H_2_O solubility of TAIC, as a confirming manifestation for the lowest *E_mix_* and *χ* of TAIC, as shown in Figure 5.

### 2.5. Initiation and Growth Mechanism of Water-Tree

The water-tree resistance of polyethylene materials is essentially determined by three attributes: (1) Except for the mechanical damage of water molecules to the amorphous phase, the extrusion and slippage of adjacent lamellae cause the expansion of amorphous phase area to form large water-filled pores, which means that the growth of water-filled pores will be retarded by intensifying the molecular connections between lamellae; (2) because water molecules are impacting and assembling in the amorphous phase of polyethylene, the initiation and growth of water micro-cracks can be inhibited by increasing the tenacity of the amorphous phase, which dominates the toughness of polyethylene materials; and (3) as the auxiliary crosslinkers in XLPE are chemically connected to the macromolecular chain of polyethylene, the micro-water beads at the insulation defects will not agglomerate into a large area due to the favorable compatibility of crosslinker and water, thus reducing the damage of micro-water beads to the amorphous region.

**Figure 7:**
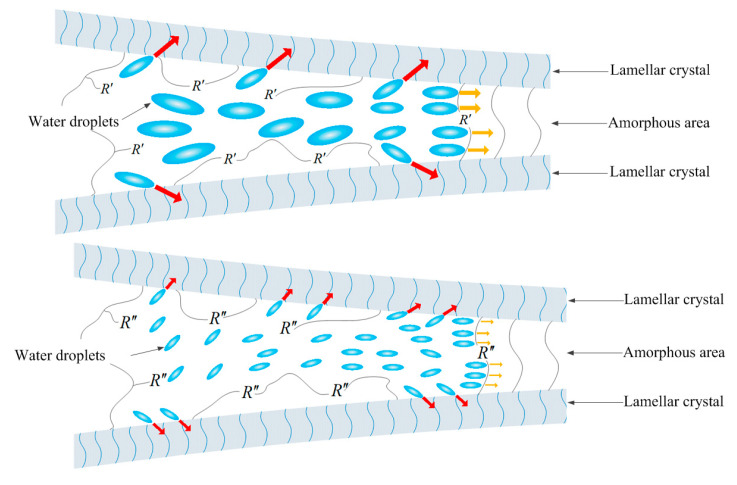
Schematic electromechanical mechanism of water microbeads diffusion and breaking through the amorphous areas in XLPE-HAV2 (top panel) and XLPE-TMPTMA or XLPE-TAIC (bottom panel).

Based on the strain hardening process and dynamic thermomechanical performances, it is reasonably proposed that the auxiliary crosslinker can be used to improve the water-resistance of XLPE materials not only by facilitating the generation of the macromolecular network structures, but also by employing its hydrophilicity to disperse micro-water molecule agglomeration. In comparison for the three UV-XLPE materials, XLPE-HAV2, with the highest strain hardening strength and *α* relaxation peaks, evidently represents the lowest water-tree resistant ability due to the lowest compatibility with water which accounts for the strong destructive ability of the micro-water beads agglomerated in a large area of amorphous phase, as schematically shown in Figure 7 where *R*′ and *R*″ denote the bridged crosslinkers of HAV2 and TAIC (or TMPTMA) respectively. Compared with XLPE-HAV2, the microbeads in XLPE-TAIC or XLPE-TMPTMA prefer to disperse into smaller water droplets with lower stresses on the molecular chains in amorphous regions between lamellae, which will barely agglomerate to form water-filled pores and lead to smaller water-trees.

It is further noted that the diffusivity of water in TMPTMA is slightly higher than in TAIC, and the strain hardening strength and *α* relaxation peak of XLPE-TMPTMA are also higher than those of XLPE-TAIC. However, the water-tree of XLPE-TAIC is distinctly smaller than that of XLPE-TMPTMA, which can be attributed to the larger number of crosslinkers in XLPE-TAIC. According to the molecular weights of 249.3 and 338.4 for TAIC and TMPTMA, respectively, the molecular number of TAIC is nearly 36% greater than that of TMPTMA for preparing UV-XLPE samples with the auxiliary crosslinkers in same mass fraction. Therefore, XLPE-TAIC with more crosslinker molecules can prevent more micro-water beads from agglomerating in the large area and thus further reduce the growth rate of water-trees.

## 3. Experimental and Theoretical Schemes

### 3.1. Material Preparation

The melt blending and hot-pressing approaches are utilized for preparing UV-initiated XLPE insulating cable materials with the raw materials being adopted as follows; linear low-density polyethylene (LLDPE, DFDA-7042, Sinopec Co. Ltd., Beijing, China) as parent material, benzophenone (BP, Jinleiyuan Chemical Co. Ltd., Lianyungang, China) as photon-initiator for initiating crosslinking reaction under UV irradiation, auxiliary crosslinkers of triallyl isocyanurate (TAIC), trimethylolpropane trimethacrylate (TMPTMA), and N,N′-m-Phenylenedimaleimide (HAV2) (Sinopharm Chemical Reagent Co. Ltd., Shanghai, China), all of purity higher than 95%. The molecular structures of the auxiliary crosslinkers are shown in Table 3. In the melting blending process for preparing initial mixtures, the pristine LLDPE are melted homogeneously in Torque Rheometer (RM200C, Hapro Co. Ltd., Harbin, China) for 3 min at 160 °C with a stirring rate of 60 rpm, and then 2 wt% BP photon-initiator and 1 wt % auxiliary crosslinkers are added to be blended for 3 min and cooled down to ambient temperature so as to obtain the uniformly mixed blend materials.

**Figure 8:**
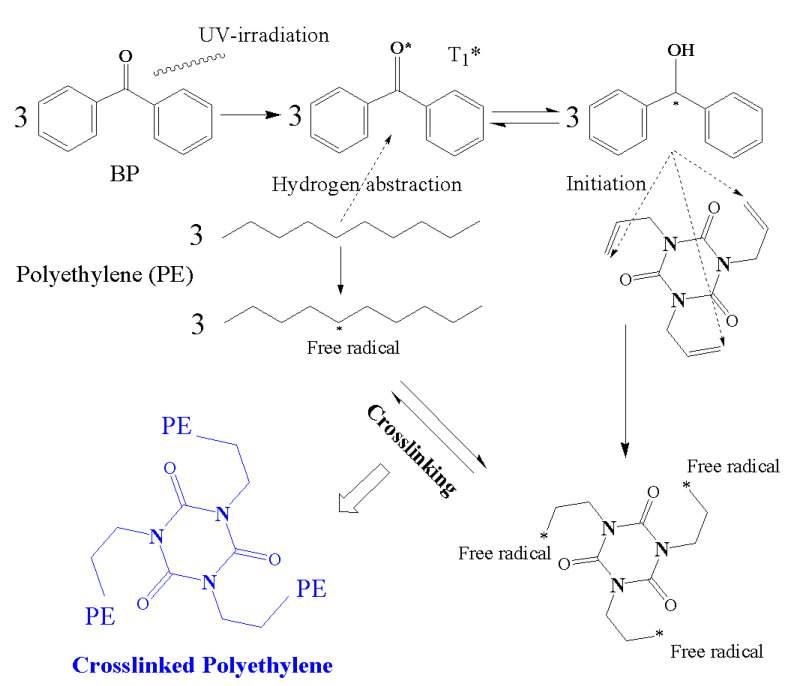
Schematic illustration of ultraviolet (UV)-initiated crosslinking reactions employing auxiliary crosslinkers (TAIC for example).

For photon-initiated crosslinking process, the prepared hot-pressed mixture is firstly put into a plate vulcanizer at 160 °C with the pressure being increased from 0 to 15 MPa by 5 MPa per 5 min approaching a completely melting state, and then irradiated by a UV source array of light-emitting diode (LED) units (NVSU233A-U365, Riya Electronics Chemistry Co., Ltd., Shanghai, China) for 10 s on an irradiation platform at normal pressure and temperature in air atmosphere. Exploiting the transparency of the LLDPE fluid at a temperature higher than the fusion point after being squeezed out from the plate vulcanizer, the UV lights are incident through the melting mixture of LLDPE, BP, and auxiliary crosslinkers. Hydrogen abstractions from polyethylene molecules to UV-excited first-triplet (T_1_) BP and the partial fractures of carbon double bonds on auxiliary crosslinker molecules are simultaneously initiated by instantaneous UV-irradiation, resulting in amounts of transient free radicals on both polyethylene and auxiliary crosslinker molecules, which are forming chemical bonds to accomplish crosslinking reactions, as schematically illustrated in Figure 8. By using auxiliary crosslinkers, the free radicals on polyethylene molecules connect though auxiliary crosslinkers to form reticulated crosslinking systems, which are nominated, respectively, as XLPE-TAIC, XLPE-TMPTMA, and XLPE-HAV2. The UV-initiated XLPE materials with auxiliary crosslinkers are finally achieved after being degassed in short-circuit at 80 °C for 48 h in a vacuum oven to eliminate the residual impurities of small molecules. In photon-initiated crosslinking process under UV irradiation, the power, wavelength, and incident direction of UV light are controlled on 1.0 W, 365 nm, and 60° angle, respectively.

### 3.2. Accelerated Water-Tree Aging Experiment

**Figure 9:**
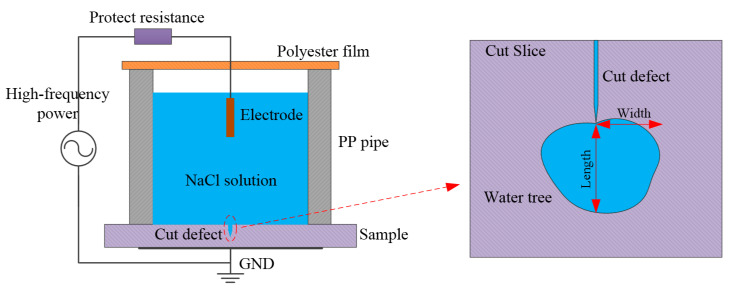
Schematic experimental set-up of water-tree initiation with water blade electrode.

The accelerated water-tree aging experiments are performed with a water blade electrode which can efficiently produce water-trees in a clear morphology, as schematically shown in Figure 9. The knife-like defect is caused at the edge of the blade electrode by cutting the conductive metal blade with a curvature radius of 0.01 mm and a thickness of 0.03 mm vertically into the side surface of the cuboid sample in a length of 100 mm and a thickness of 4 mm, with a distance of 2 mm being kept from the blade edge to the other side of cuboid surface. The water medium of 1.8 mol/L sodium chloride solution is used for water-tree aging experiments. To increase the initiation probability and growth rate of water-trees with appreciable morphology characteristics for different materials, the AC high voltage power supply with a frequency of 3 kHz and an effective value of 4 kV is adopted to continuously apply on samples for 7 days. Before applying voltage, all of the equipment for the water-tree experiments is placed in a vacuum environment, being kept for 30 min to fully eliminate the residual air in blade edge defects. When the water-tree experiments of applying voltage is finished, the defects at the blade edge are cut along the longitudinal direction of the cuboid sample into thin slices with a thickness of 120 μm by a manual rotary microtome (Leica RM2235, Shanghai Chuangxun Medical Equipment Co. Ltd., Shanghai, China). Finally, the slice samples are impregnated in methylene blue solution for 4 h at the temperature of 90 °C.

### 3.3. Characterization and Measurement

The thermal extension test is performed with the sample preparation and measurement procedures being in accordance with GB/T 2951.11-2008. First, the dumbbell-shaped specimens with a thickness of 1 mm and a middle scaled distance of 20 mm are prepared. Then, the specimens are fixed and hung vertically and weighted according to the standard of 0.2 N/mm^2^. Finally, the specimens are placed in the environment of 200 °C for 15 min. The gauge length is recorded to calculate load elongation. A set of experiments is carried out to calculate the average.

Gel content tests are carried out by the solvent extraction method according to the standard of ASTM D 2765-2011. The gel extraction experiments are carried out using xylene solvent in the reaction container of three flasks. The thermometer at the side mouth of the flask is utilized to monitor the solvent temperature in real-time so that the xylene solvent can be controlled in a slightly boiling state. The gel extraction experiment is specialized as follows; (a) material sample is weighed as M_1_ in mass and placed in a square (45 mm side length) mesh pocket made from stainless steel filter screen, and then the total mass of the sample and mesh pocket is weighted as M_2_; (b) the stainless steel mesh is connected with aluminum wire and immersed into xylene solution; (c) the electronic heating device is operated to control the temperature at 130–140 °C so that the xylene solution is heated to boiling and the condensate tube can drop ~20 water droplets per minute; (d) after 12 h, the stainless steel mesh pocket is taken out from xylene solvent and placed it into an oven at 80 °C for 12 h to completely evaporate the residual xylene solvent; and (e) finally, the stainless steel mesh pocket is removed and weighed as M_3_ in mass to calculate gel content of the sample by the equation (M_3_-M_2_)/M_1_.

Conforming to the standard of GB/T 1040.2-2006, the stress–strain characteristics are tested with an elongation speed of 5 mm/min for the samples being fabricated into a “5A” dumbbell shape with a thickness of 1 mm and a mark distance of 20 mm. The dynamic thermomechanical analysis (DMA) is employed to evaluate the viscoelasticity of the samples in a dimension of 80 × 10 × 1 mm by measuring the energy-storage modulus *E*′, loss modulus *E*″, and loss factor tan*θ = E*″/*E*′; in the temperature range of −50 to 150 °C, as implemented by a dynamic thermomechanical analyzer (Q800DMA, TA apparatus Co. Ltd., Delaware, USA) with a heating rate of 3 °C/min in nitrogen atmosphere. The DMA tests are implemented under tensile mode with the target amplitude, frequency, static force, and dynamic force being set as 15 μm, 1 Hz, 0.375, and 0.3 N, respectively.

### 3.4. Miscibility Calculation Method

The molecular interaction binding energy, free energy, and phase diagram of water (H_2_O) and auxiliary crosslinker (AC) mixtures are calculated by Monte Carlo molecular simulation technique combined with the modified Flory–Huggins model, as implemented in Blends module of Materials Studio 8.0 package, to investigate the compatibility of H_2_O and AC [31,32]. The simplest and best-known theory of the thermodynamics of mixing and phase separation in binary systems is the Flory–Huggins model. The general expression for the free energy of mixing of a binary system is

(3) $$\frac{\Delta G}{RT}=\frac{\phi_{b}}{n_{b}}\ln\phi_{b}+\frac{\phi_{s}}{n_{s}}\ln\phi_{s}+\chi \phi_{b}\phi_{s}$$

where Δ*G* denotes the free energy of mixing (per mole), *ϕ*_i_ represents the volume fraction of component i, *n*_i_ signifies the degree of polymerization of component i, *χ* symbolizes the interaction parameter, *T* is the absolute temperature, and *R* is the gas constant. The first two terms represent the combinatorial entropy, which is always negative, thus favoring a mixed state over the pure components. The last term is the interaction free energy. If the interaction parameter *χ* is positive, this term disfavors a mixed state. The interaction parameter defined as *χ* = *E*_mix_/*RT* (*E*_mix_ is the mixing energy) implies the difference in free energy due to interaction between the mixed and pure states.

## 4. Conclusions

The water-tree resistances of UV-XLPE materials prepared with different auxiliary crosslinkers are studied with the water blade electrode method to reveal the initiation and growth mechanisms of water-trees in correlation with the hydrophilicity of auxiliary crosslinkers. The amorphous phase tenacity, the strain hardening strength, and the compatibility of crosslinker and water are investigated by mechanical tests and molecular simulations to elucidate the hydrophilicity of auxiliary crosslinkers affecting on the water resistability of UV-XLPE. For the same crosslinker content, XLPE-TAIC and XLPE-HAV2 exhibit the highest and the lowest water-tree resistances, respectively, which is consistent with them having the lowest and highest miscibilities. It is suggested that the promotion of water-tree resistance by raising hydrophilicity of auxiliary crosslinker is attributed to the preferred diffusion of water molecules through auxiliary crosslinkers. The large number of TAIC molecules in XLPE-TAIC and the low mixing energy and interaction parameter of TAIC and water account for the excellent water resistibility of XLPE-TAIC.

## Figures and Tables

**Table 1:** Thermal elongation and gel content.

**Samples**	**Thermal Elongation/%**	**Gel Content/%**
XLPE-TMPTMA	40	88
XLPE-TAIC	45	85
XLPE-HAV2	30	91

**Table 2:** Water-tree dimension and *β* relaxation parameter.

**Samples**	**Loss Modulus** **/MPa**	**Loss Factor tan*θ***	**Characteristic Length/μm**	**Characteristic Width/μm**
LLDPE	77	0.076	322.8	312.5
XLPE-TMPTMA	82	0.084	240.4	191.4
XLPE-TAIC	86	0.080	195.9	146.2
XLPE-HAV2	89	0.089	263.7	225.1

**Table 3:** Molecular structures of auxiliary crosslinkers.

**A** **bbreviation**	**TAIC**	**TMPTMA**	**HAV2**
Molecular structure	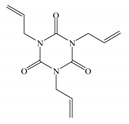	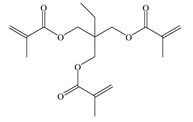	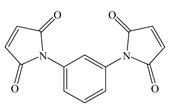
Chemical formula	C_12_H_15_N_3_O_3_	C_18_H_26_O_6_	C_14_H_8_N_2_O_4_
